# Characterization of HPV DNA methylation of contiguous CpG sites by bisulfite treatment and massively parallel sequencing—the FRAGMENT approach

**DOI:** 10.3389/fgene.2014.00150

**Published:** 2014-06-03

**Authors:** Chang Sun, Thomas McAndrew, Benjamin C. Smith, Zigui Chen, Marina Frimer, Robert D. Burk

**Affiliations:** ^1^Department of Pediatrics, Albert Einstein College of MedicineBronx, NY, USA; ^2^Department of Obstetrics, Gynecology and Women's Health, Albert Einstein College of MedicineBronx, NY, USA; ^3^Department of Microbiology and Immunology, Albert Einstein College of MedicineBronx, NY, USA; ^4^Department of Epidemiology and Population Health, Albert Einstein College of MedicineBronx, NY, USA

**Keywords:** human papillomavirus, methylation, next generation sequencing, CpG methylation, methylation haplotypes

## Abstract

Invasive cervix cancer (ICC) is the third most common malignant tumor in women and human papillomavirus 16 (HPV16) causes more than 50% of ICC. DNA methylation is a covalent modification predominantly occurring at CpG dinucleotides and increased methylation across the HPV16 genome is strongly associated with ICC development. Next generation (Next Gen) sequencing has been proposed as a novel approach to determine DNA methylation. However, utilization of this method to survey CpG methylation in the HPV16 genome is not well described. Moreover, it provides additional information on methylation “haplotypes.” In the current study, we chose 12 random samples, amplified multiple segments in the HPV16 bisulfite treated genome with specific barcodes, inspected the methylation ratio at 31 CpG sites for all samples using Illumina sequencing, and compared the results with quantitative pyrosequencing. Most of the CpG sites were highly consistent between the two approaches (overall correlation, *r* = 0.92), thus verifying that Next Gen sequencing is an accurate and convenient method to survey HPV16 methylation and thus can be used in clinical samples for risk assessment. Moreover, the CpG methylation patterns (methylation haplotypes) in single molecules identified an excess of complete-and non-methylated molecules and a substantial amount of partial-methylated ones, thus indicating a complex dynamic for the mechanisms of HPV16 CpG methylation. In summary, the advantages of Next Gen sequencing compared to pyrosequencing for HPV genome methylation analyses include higher throughput, increased resolution, and improved efficiency of time and resources.

## Introduction

Invasive cervical cancer (ICC) is the third most common malignant tumor in women and is caused by persistent infection of oncogenic human papillomavirus (HPV) (Jemal et al., 2011), especially type 16, which accounts for greater than 50% of all ICC (Schiffman et al., 2007; Li et al., 2011; Schiffman and Wentzensen, 2013). Recent data indicates that multiple regions of HPV16 and other oncogenic HPV type genomes show increasing CpG methylation patterns among normal, cervical intraepithelial neoplasia (CIN), and cancer tissues, respectively (Badal et al., 2003; Kalantari et al., 2004, 2009, 2010, 2014; Hong et al., 2008; Brandsma et al., 2009, 2014; Ding et al., 2009; Fernandez et al., 2009; Fernandez and Esteller, 2010; Piyathilake et al., 2011; Sun et al., 2011; Wentzensen et al., 2012; Lorincz et al., 2013; Mirabello et al., 2013). Thus, assays for quantitation of CpG methylation of oncogenic HPV genomes in general and HPV16 in particular, indicate that methylation is a promising biomarker for ICC development (Clarke et al., 2012). Therefore, a fast, accurate, and high-throughput approach to survey DNA methylation in HPV16 should facilitate ICC prevention, diagnosis, and treatment.

So far, the most widely used method for HPV DNA methylation investigation is bisulfite treatment followed by sequencing (Bhattacharjee and Sengupta, 2006), MassArray (Ehrich et al., 2005), SNPshot (Kaminsky and Petronis, 2009), or in particular pyrosequencing (Tost and Gut, 2007a,b; Dejeux et al., 2009) (for review, see Clarke et al., 2012). Despite the accuracy of CpG quantitation by pyrosequencing, it can only provide a relatively short read for each assay per sample and thus is time and labor intensive for testing multiple sites in large numbers of samples, which limits the incorporation of DNA methylation in clinical studies. Moreover, all these approaches constrain the ability to detect the methylation pattern at single-DNA-molecule resolution, which is critical for investigating methyltransferase dynamics. Although the cloning-sequencing approach after bisulfite treatment can provide some insight into this issue, the typically low number of clones analyzed (<10) and the high costs limits this approach.

Next generation sequencing can yield millions of single molecule reads and has been used to determine DNA methylation (Taylor et al., 2007; Bibikova and Fan, 2010; Laird, 2010; Feng et al., 2011; Kim et al., 2011; Komori et al., 2011; Ku et al., 2011; Nejman et al., 2014). Supplemented with DNA barcoding technology, which incorporates a unique index sequence into each PCR segment, this approach can provide a rapid way to simultaneously determine DNA methylation at the single-molecule level in large numbers of samples. However, the accuracy and validity of this approach needs further evaluation, especially in viral genomes such as HPV16.

In the present study, we randomly chose 12 samples with quantitation of CpG methylation within the HPV16 genome by pyrosequencing and performed amplification with primers containing barcodes specific for each sample. After all samples were pooled and purified, the PCR products were deep sequenced, analyzed, and the results were compared with CpG methylation determined by pyrosequencing. The methylation ratio for most CpG sites was highly correlated with those from pyrosequencing, which indicated that Next Gen sequencing of bisulfite treated cervical cells infected with HPV16 was an accurate method of quantitating CpG methylation. Moreover, the single molecule analyses provides a “methylation haplotype” and indicated an excess of full and non-methylated molecules in nearly all samples and a lower proportion of partially methylated molecules in most samples, thus revealing a complex and mosaic methylation pattern in the HPV16 genome.

## Materials and methods

### Cervicovaginal samples

Twelve exfoliated cervical samples were randomly chosen from a previously reported nested case-control study of HPV16-positive cervical intraepithelial neoplasia grade 3 (CIN3) and HPV16-positive cervical samples that cleared infection (Mirabello et al., 2012). The lab was blinded to all clinical information. All samples contained HPV16 and the quantitation of CpG methylation of the HPV16 genome had been determined by pyrosequencing, as described (Mirabello et al., 2012). The study was designed to test samples for quantitation of CpG methylation to evaluate Next Gen sequencing compared to pyrosequencing prior to embarking on using this method on precious well-characterized samples from epidemiological studies.

### Assay design

**Figure 1:**
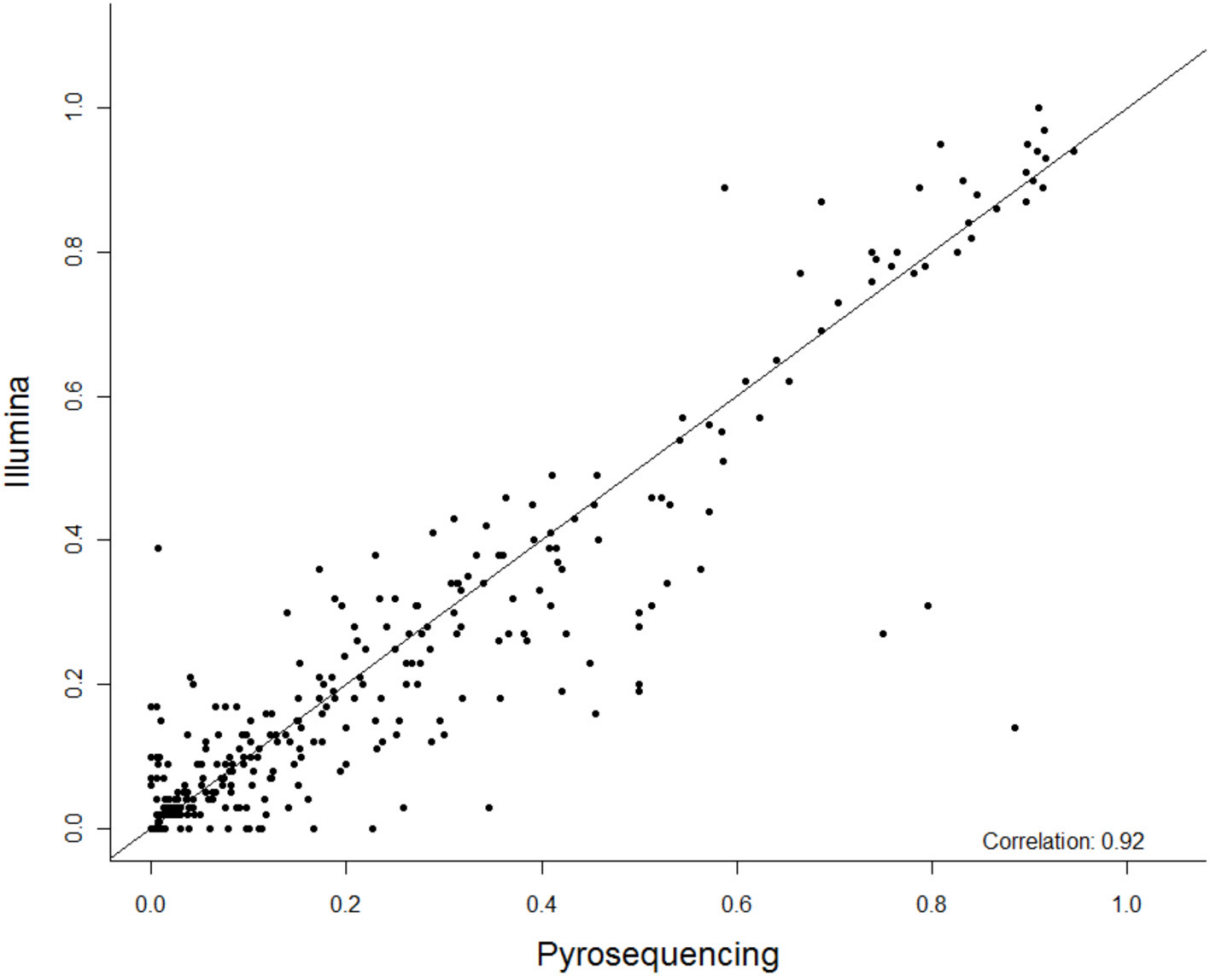
**The summary correlation of 27 CpG methylation sites between pyrosequencing and Next Gen sequencing**. The x-axis is percent methylation by pyrosequencing and the y-axis is percent methylation by Next Gen sequencing. Each CpG site is plotted for each subject.

Since L1, L2, and E2 ORF regions showed differential methylation among disease groups (Mirabello et al., 2012), these three regions and the most significant CpG sites within them were chosen for the current study. Primers for PCR were designed using MethPrimer (Li and Dahiya, 2002) (http://www.urogene.org/methprimer/index1.html). In total, 3 segments in L1, 4 in L2, and 1 in E2 were included in the current study (Table 1A), and in total, 31 CpG sites were surveyed. Each primer was labeled by a unique barcode and 5′ and 3′ padding sequence (Table 1B) and synthesized by Integrated DNA Technologies (IDT, Coralville, IA). A map of all 112 CpG sites in the HPV16 7906 bp reference genome is shown in Figure 1 in the review by Clarke et al. (2012).

**Table 1A:** **Next Gen sequencing assays of bisulfite treated HPV16 DNA in clinical samples**.

**Assay name**	**#CpG**	**CpG position**	**Length (bp)**
L1_1	4	5602, 5608, 5611, 5617	114
L1_2	4	7034, 7091, 7136, 7145	172
L1_7	2	6650, 6581	167
L2_1	5	4240, 4249, 4261, 4270, 4277	130
L2_2	3	4427, 4437, 4441	89
L2_4	3	5128, 5173, 5179	123
L2_5	1	5378	166
E2_1	5	3412, 3415, 3417, 3433, 3436 (*3448, 3462, 3473, 3496*)	169

*( ), These sites were present in the NGS data but not PSQ*.

**Table 1B:** Description of barcoded primers^*^ for assays shown in this table.

**Primer name**	**5′ pad (LP)**	**3′ pad (RP)**	**Primer target sequence (5′-3′)**
16E2_1F	ACT	GCAG	TTAGGTAGTATTTGGTTAATTATTT
16E2_1R	ACT	GCAG	ATTAAAACACTATCCACTAAATCTCTATAC
16L1_1F	TAC	GTAC	TAATATATAATTATTGTTGATGTAGGTGAT
16L1_1R	TAC	GTAC	AACAACCAAAAAAACATCTAAAAAA
16L1_2F	ACT	GACG	TTTGTAGATTTAGATTAGTTTTTTTTAGGA
16L1_2R	ACT	GACG	TTCAACATACATACAATACTTACAACTTAC
16L1_7F	TAC	GATG	ATGTAGTTTTTGAAGTAGATATGGTAGTA
16L1_7R	TAC	GATG	AATTACCTCTAATACCCAAATATTCAA
16L2_1F	ATC	GACG	TTTTTGTTTGTTTGTTTGTTTTT
16L2_1R	ATC	GACG	ACATATACCTACCTATTTACATATTTTATA
16L2_2F	ATC	GACG	TATGGAAGTATGGGTGTATTTTT
16L2_2R	ATC	GACG	ATTCCCAATAAAATATACCCAATAC
16L2_4F	ATC	GTAC	TTTTGGATATAGTTGTTTTATATAGGTTAG
16L2_4R	ATC	GTAC	CCTTAACACCTATAAATTTTCCACTAC
16L2_5F	ATC	GTCA	TTGTAGAAGAAATAGAATTATAAATTATAA
16L2_5R	ATC	GTCA	AAAAATATAAAAAATACAAATAATACC

* *Each primer consisted of 5′ to 3′: 3 bp (LP) – 8 bp Barcode – 4 bp (RP) – Primer Target Sequence*.

### Bisulfite treatment, PCR, purification, and deep sequencing

DNA samples containing HPV16 DNA were treated with freshly prepared bisulfite using the EZ DNA methylation kit (Zymo Research, Orange, CA) according to the manufacturer's protocol. Upon bisulfite treatment, unmethylated C's are converted into U's, which are then converted to T's by Taq polymerase during PCR amplification; methylated C's remain unmodified. Thus, in the CpG sequence a “C” represents a methylated CpG, whereas a “T” represents an unmethyaled CpG. All segments were amplified by HotStart-IT FideliTaq DNA polymerase (United States Biochemicals, Cleveland, OH). After validating size and intensity in a 3% agarose gel, each PCR product was pooled in equal proportions, separated by electrophoresis, and isolated from the gel. After precipitation by isopropanol and washed by 70% ethanol, the PCR products were ligated with adaptor (library construction) and sequenced on an Illumina HiSeq 2000 (NG sequencing) within the Albert Einstein College of Medicine, Epigenetics Core Facility (Bronx, NY).

### Methylation ratio analysis

The obtained sequences were filtered by prinseq (Schmieder and Edwards, 2011) with average Phred score (Cock et al., 2010) not less than 20. The barcodes for each sample were split and cut by FastX kit (http://hannonlab.cshl.edu/fastx_toolkit/index.html). After alignment with the reference HPV16 genome by bowtie (Langmead et al., 2009), the methylation status for each molecule was determined by Bismark (Krueger and Andrews, 2011). For each CpG site, the methylation ratio is calculated by the formula: number of C reads divided by the sum of C and T reads at each CpG site. The pyrosequencing result for each site was determined on a PSQ96 ID Pyrosequencer (Qiagen, Valencia, CA) at the Albert Einstein College of Medicine, Genomics Core Facility (Bronx, NY) and the readout was percent methylation, as previously described (Mirabello et al., 2012). The correlation between NG sequencing and pyrosequencing was performed by linear regression in SPSS 16.0 (SPSS Inc., Chicago, IL) and the null hypothesis was rejected when *P* < 0.05. We have deposited the read sequences in the NCBI Sequence Read Archive (SRA) database, accession number SRP040981.

### Single molecule analysis

The methylation pattern for each single molecule and the counts of each pattern were obtained by an in-house script (available on request). Briefly, the Bismark output, which gave the methylation state for each CpG site in each molecule, was parsed, and the methylation pattern of each DNA molecule was then reconstructed based on the unique read name it was assigned by the sequencer. Finally, the prevalence of each unique methylation pattern was counted, for each sample in each assay. The expected probabilities were constructed in two steps. First the singular probabilities of each site being methylated and unmethylated were calculated, by counting the proportions of molecules in each state at each site. Multiplying the appropriate singular probabilities, under the assumption that CpG sites would be methylated independently, produced an expected probability for each methylation pattern. A χ^2^ goodness of fit test was then performed to compare observed and expected probabilities for methylation patterns, where the observed probabilities were the proportions of each detected pattern, calculated from their counts.

## Results

### Sequencing data statistics

**Figure 2:**
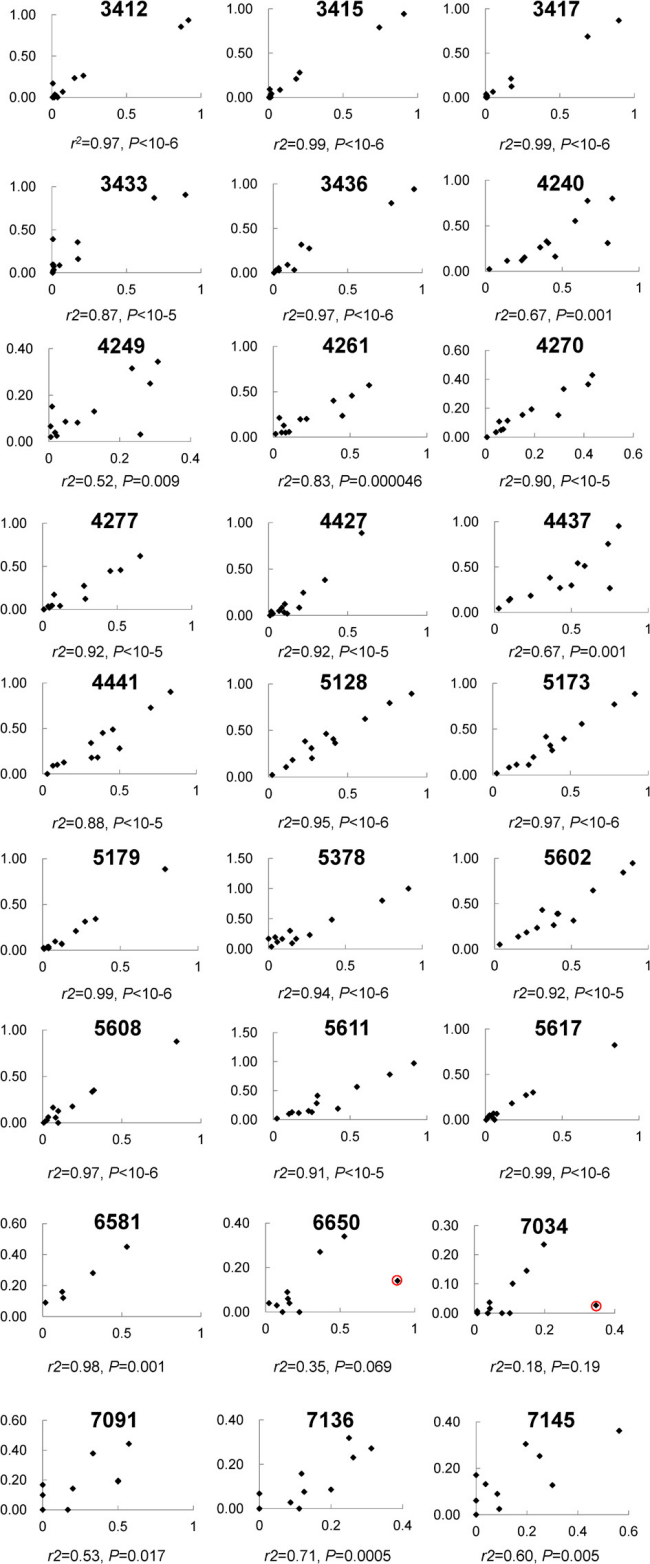
**The correlation of 27 individual CpG methylation sites between pyrosequencing and Next Gen sequencing**. Each plot displays one CpG site and the location is indicated at the top. The x-axis indicates Illumina sequencing, the y-axis pyrosequencing for percent methylation.

In total, 192.2 million reads 95 bp long were obtained from NG sequencing and 53.4 million (27.8%) possessed an average Phred score above 20 and were used for this analysis (Ewing and Green, 1998). 41.7 million reads (78.1%) were observed to contain one of the incorporated barcodes without mismatch and assigned to a corresponding sample for further analysis. Except one segment in the L2 gene, most segments included ~4–8 million reads (Figure S1A). For each sample, the read count varied from 2 to 6 million (Figure S1B). Most CpG sites (21/27) were covered by 0.6 to 1.8 million reads (Figure S1C), in total. Although three fragments did not amplify as robustly and had less reads (i.e., L1_2, L2_2, and L2_5 assays containing CpG sites 7034, 7091, 7136, 7145; 4427, 4437, 4441; and 5378, respectively), the correlation between CpG sites within these fragments between the PSQ and NGS assays had reasonable agreement (see Figure 2).

### Methodological comparison

Among these 31 CpG sites, 27 had been analyzed by pyrosequencng and the two results were compared. Using linear regression, most sites showed significant correlation between the two methods with an overall correlation of 0.92 (Figure 1). Only two CpG sites (positions 6650 and 7034) were poorly correlated (*P* = 0.069 and 0.19, respectively, Figure 2). These results indicated that next generation sequencing was an appropriate method for determining CpG methylation in the HPV16 genome, yielding results that were highly correlated with a well-established pyrosequencing technique.

### Single-molecule resolution of CPG methylation

**Figure 3:**
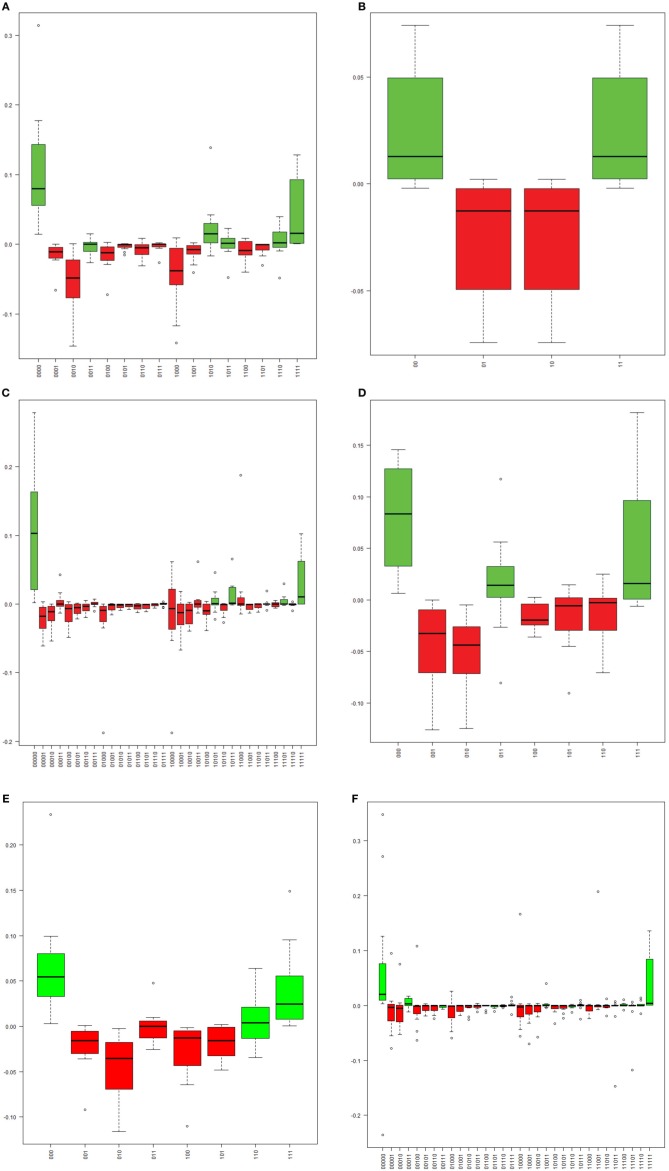
**The distribution of the differences between observed and expected frequencies for each “methylation haplotype” in assay L1_1 (A), L1_7 (B), L2_1 (C), L2_2 (D), L2_4 (E), and E2_1 (F)**. Each boxplot indicate one “methylation haplotype” combination. The green and red bars denote positive and negative value, respectively. **(A)** “Methylation haplotype” frequency differences in assay L1_1. **(B)** “Methylation haplotype” frequency differences in assay L1_7. **(C)** “Methylation haplotype” frequency differences in assay L2_1. **(D)** “Methylation haplotype” frequency differences in assay L2_2. **(E)** “Methylation haplotype” frequency differences in assay L2_4. **(F)** “Methylation haplotype” frequency differences in assay E2_1.

The methylation pattern for each single molecule was determined for each sample across six regions of the HPV16 genome. A substantial proportion (10–80%) of molecules in the six assays were partially methylated (possessing a mixture of methylated and unmethylated sites), and the distributions of patterns were varied. However, the site-wise proportions of methylation for a given sample in a given assay were similar and the distribution of patterns in each molecule did exhibit dependence on that methylation level. To address this issue, we calculated the expected frequencies of all possible methylation patterns in each assay and compared them with the observed patterns (Figure 3). In most samples, a relative excess of none- and/or fully methylated molecules was observed (Figure 3). In contrast, despite their high prevalences, there was a relative absence of partial methylated molecules (Figure 3). As a consequence, most of the samples yielded a significant *P*-value (*p* < 0.05), thus indicating that CpG sites are not likely to be methylated/demethylated in an independent fashion, but that a more complex process determines the methylation state within a region of the HPV16 genome.

## Discussion

In the present study, we used Illumina Next Gen single molecule sequencing to survey the methylation of the HPV16 genome in 12 samples and compared the result with pyrosequencing, which provides an average percent methylation for each CpG site. A consequence of using Next Gen sequencing technology was the ability to investigate the methylation pattern at the single-molecule level. Our results demonstrate that the Next Gen bisulfite sequencing protocol was comparable to the well-established pyrosequencing-based method for assaying HPV16 genome methylation (overall correlation = 0.92). The ability to analyze single molecules allowed us to test whether the process of CpG methylation at specific sites was independent. Thus with known percent CpG methylation at each site we compared the observed with the predicted methylation haplotypes. There was a significant difference indicating that CpG methylation of multiple CpG sites on a given fragment of the viral genome is not an independent process. In addition, utilizing DNA barcoding, multiple samples can be pooled together and run in a single sequencing reaction and the result for each single sample can be distinguished without ambiguity. Thus, the high-throughput nature of this technique facilitates large-scale clinical and epidemiological studies.

Several CpG sites presented a relatively low read count compared with others. A careful inspection indicated that these were located in the middle of the amplicon and would thus appear at the end of the read in both strand. These end regions usually have a low base call quality, due to constraints of the sequencing technology and are, therefore, often filtered out prior to analysis. The Bismark-based analysis method utilized in this study doesn't exploit the gain in base call quality that can be obtained by comparing overlapping regions of paired-end reads, therefore the latter problem could be surmounted by improvements in the bioinformatics pipeline. Alternatively, we could shorten the amplicon, or generate longer reads, to facilitate equal read counts for all sites in an assay. However, there are limitations on shortening or lengthening the amplicons, due to the composition of sequences surrounding CpG sites that are used for the primers. Nevertheless, it is anticipated that with advances in Next Gen sequencing technologies longer fragment reads and improved quality will facilitate the use of the methods described in this report.

Despite the high consistency between next generation sequencing and pyrosequencing results, two CpG sites, 6650 and 7034, failed to yield a significant correlation. Both CpG sites had a relatively high read count (>1.14 and 0.15 million, respectively), thus indicating that the low correlation was not due to stochastic effects. A detailed audit identified one sample showing remarkable discrepancy between the two approaches in both CpG sites (see red circle in Figure 2). When this sample was removed from analysis, significant correlations were obtained for both CpG sites (*r*^2^ = 0.85, *P* = 0.00006 for 6650 and *r*^2^ = 0.76, *P* = 0.001 for 7034), which further verified the strong consistency between the two approaches. However, the reason for the discrepancy in this sample remains unclear. Possibilities include a nucleotide variation at this position, which would skew the results, and/or bisulfite- and PCR-induced artifacts. In addition, potential PCR biases could also result in lower than expected read numbers.

Examining methylation patterns on individual molecules of DNA is essential to understand the methyltransferase dynamics and the mechanism(s) by which methylation interacts with oncogenic HPV viral natural history and progression to cervical cancer. Based on cloning approaches, most previous studies suggested that methylation in promoter regions was more likely to be one (fully methylated) or zero (fully unmethylated) in human genomic DNA (Oates et al., 2006) and HPV genomes (Kalantari et al., 2004, 2009, 2010; Turan et al., 2006, 2007; Ding et al., 2009; Fernandez et al., 2009). However, due to the low number of single molecules analyzed (i.e., clones sequenced), the real composition might be substantially different. Next Gen sequencing, which can survey millions of molecules at the same time, can provide more insight into this issue. In our results, despite the relative excess of fully methylated and fully unmethylated molecules, a substantial proportion of molecules displayed a partial methylation pattern, which verified previous observations (Taylor et al., 2007) and hinted at the complex regulation of the methylation process. Whether there are dynamic changes in CpG methylation patterns remains to be determined.

### Conflict of interest statement

The authors declare that the research was conducted in the absence of any commercial or financial relationships that could be construed as a potential conflict of interest.

## References

[B1] Badal V. Chuang L. S. Tan E. H. Badal S. Villa L. L. Wheeler C. M. (2003). CpG methylation of human papillomavirus type 16 DNA in cervical cancer cell lines and in clinical specimens: genomic hypomethylation correlates with carcinogenic progression. J. Virol. 77, 6227–6234 10.1128/JVI.77.11.6227-6234.2003 12743279 PMC154984

[B2] Bhattacharjee B. Sengupta S. (2006). CpG methylation of HPV 16 LCR at E2 binding site proximal to P97 is associated with cervical cancer in presence of intact E2. Virology 354, 280–285 10.1016/j.virol.2006.06.018 16905170

[B3] Bibikova M. Fan J. B. (2010). Genome-wide DNA methylation profiling. Wiley Interdiscip. Rev. Syst. Biol. Med. 2, 210–223 10.1002/wsbm.35 20836023

[B4] Brandsma J. L. Harigopal M. Kiviat N. B. Sun Y. Deng Y. Zelterman D. (2014). Methylation of twelve CpGs in human papillomavirus type 16 (HPV16) as an informative biomarker for the triage of women positive for HPV16 infection. Cancer Prev. Res. (Phila.) 7, 526–533 10.1158/1940-6207.CAPR-13-0354 24556390

[B5] Brandsma J. L. Sun Y. Lizardi P. M. Tuck D. P. Zelterman D. Haines G. K. 3rd. (2009). Distinct human papillomavirus type 16 methylomes in cervical cells at different stages of premalignancy. Virology 389, 100–107 10.1016/j.virol.2009.03.029 19443004 PMC2918277

[B6] Clarke M. A. Wentzensen N. Mirabello L. Ghosh A. Wacholder S. Harari A. (2012). Human papillomavirus DNA methylation as a potential biomarker for cervical cancer. Cancer Epidemiol. Biomarkers Prev. 21, 2125–2137 10.1158/1055-9965.EPI-12-0905 23035178 PMC3664203

[B7] Cock P. J. Fields C. J. Goto N. Heuer M. L. Rice P. M. (2010). The Sanger FASTQ file format for sequences with quality scores, and the Solexa/Illumina FASTQ variants. Nucleic Acids Res. 38, 1767–1771 10.1093/nar/gkp1137 20015970 PMC2847217

[B8] Dejeux E. El abdalaoui H. Gut I. G. Tost J. (2009). Identification and quantification of differentially methylated loci by the pyrosequencing technology. Methods Mol. Biol. 507, 189–205 10.1007/978-1-59745-522-0_15 18987816

[B9] Ding D. C. Chiang M. H. Lai H. C. Hsiung C. A. Hsieh C. Y. Chu T. Y. (2009). Methylation of the long control region of HPV16 is related to the severity of cervical neoplasia. Eur. J. Obstet. Gynecol. Reprod. Biol. 147, 215–220 10.1016/j.ejogrb.2009.08.023 19819061

[B10] Ehrich M. Nelson M. R. Stanssens P. Zabeau M. Liloglou T. Xinarianos G. (2005). Quantitative high-throughput analysis of DNA methylation patterns by base-specific cleavage and mass spectrometry. Proc. Natl. Acad. Sci. U.S.A. 102, 15785–15790 10.1073/pnas.0507816102 16243968 PMC1276092

[B11] Ewing B. Green P. (1998). Base-calling of automated sequencer traces using phred. II. Error probabilities. Genome Res. 8, 186–194 10.1101/gr.8.3.175 9521922

[B12] Feng S. Rubbi L. Jacobsen S. E. Pellegrini M. (2011). Determining DNA methylation profiles using sequencing. Methods Mol. Biol. 733, 223–238 10.1007/978-1-61779-089-8_16 21431774

[B13] Fernandez A. F. Esteller M. (2010). Viral epigenomes in human tumorigenesis. Oncogene 29, 1405–1420 10.1038/onc.2009.517 20101211

[B14] Fernandez A. F. Rosales C. Lopez-Nieva P. Grana O. Ballestar E. Ropero S. (2009). The dynamic DNA methylomes of double-stranded DNA viruses associated with human cancer. Genome Res. 19, 438–451 10.1101/gr.083550.108 19208682 PMC2661803

[B15] Hong D. Ye F. Lu W. Hu Y. Wan X. Chen Y. (2008). Methylation status of the long control region of HPV 16 in clinical cervical specimens. Mol. Med. Rep. 1, 555–560 10.3892/mmr.1.4.555 21479449

[B16] Jemal A. Bray F. Center M. M. Ferlay J. Ward E. Forman D. (2011). Global cancer statistics. CA Cancer J. Clin. 61, 69–90 10.3322/caac.20107 21296855

[B17] Kalantari M. Calleja-Macias I. E. Tewari D. Hagmar B. Lie K. Barrera-Saldana H. A. (2004). Conserved methylation patterns of human papillomavirus type 16 DNA in asymptomatic infection and cervical neoplasia. J. Virol. 78, 12762–12772 10.1128/JVI.78.23.12762-12772.2004 15542628 PMC525027

[B18] Kalantari M. Chase D. M. Tewari K. S. Bernard H. U. (2010). Recombination of human papillomavirus-16 and host DNA in exfoliated cervical cells: a pilot study of L1 gene methylation and chromosomal integration as biomarkers of carcinogenic progression. J. Med. Virol. 82, 311–320 10.1002/jmv.21676 20029805

[B19] Kalantari M. Garcia-Carranca A. Morales-Vazquez C. D. Zuna R. Montiel D. P. Calleja-Macias I. E. (2009). Laser capture microdissection of cervical human papillomavirus infections: copy number of the virus in cancerous and normal tissue and heterogeneous DNA methylation. Virology 390, 261–267 10.1016/j.virol.2009.05.006 19497607 PMC2753400

[B20] Kalantari M. Osann K. Calleja-Macias I. E. Kim S. Yan B. Jordan S. (2014). Methylation of human papillomavirus 16, 18, 31, and 45 L2 and L1 genes and the cellular DAPK gene: considerations for use as biomarkers of the progression of cervical neoplasia. Virology 448C, 314–321 10.1016/j.virol.2013.10.032 24314662 PMC4051423

[B21] Kaminsky Z. Petronis A. (2009). Methylation SNaPshot: a method for the quantification of site-specific DNA methylation levels. Methods Mol. Biol. 507, 241–255 10.1007/978-1-59745-522-0_18 18987819

[B22] Kim J. H. Dhanasekaran S. M. Prensner J. R. Cao X. Robinson D. Kalyana-Sundaram S. (2011). Deep sequencing reveals distinct patterns of DNA methylation in prostate cancer. Genome Res. 21, 1028–1041 10.1101/gr.119347.110 21724842 PMC3129246

[B23] Komori H. K. Lamere S. A. Torkamani A. Hart G. T. Kotsopoulos S. Warner J. (2011). Application of microdroplet PCR for large-scale targeted bisulfite sequencing. Genome Res. 21, 1738–1745 10.1101/gr.116863.110 21757609 PMC3202290

[B24] Krueger F. Andrews S. R. (2011). Bismark: a flexible aligner and methylation caller for Bisulfite-Seq applications. Bioinformatics 27, 1571–1572 10.1093/bioinformatics/btr167 21493656 PMC3102221

[B25] Ku C. S. Naidoo N. Wu M. Soong R. (2011). Studying the epigenome using next generation sequencing. J. Med. Genet. 48, 721–730 10.1136/jmedgenet-2011-100242 21825079

[B26] Laird P. W. (2010). Principles and challenges of genomewide DNA methylation analysis. Nat. Rev. Genet. 11, 191–203 10.1038/nrg2732 20125086

[B27] Langmead B. Trapnell C. Pop M. Salzberg S. L. (2009). Ultrafast and memory-efficient alignment of short DNA sequences to the human genome. Genome Biol. 10:R25 10.1186/gb-2009-10-3-r25 19261174 PMC2690996

[B28] Li L. C. Dahiya R. (2002). MethPrimer: designing primers for methylation PCRs. Bioinformatics 18, 1427–1431 10.1093/bioinformatics/18.11.1427 12424112

[B29] Li N. Franceschi S. Howell-Jones R. Snijders P. J. Clifford G. M. (2011). Human papillomavirus type distribution in 30,848 invasive cervical cancers worldwide: variation by geographical region, histological type and year of publication. Int. J. Cancer 128, 927–935 10.1002/ijc.25396 20473886

[B30] Lorincz A. T. Brentnall A. R. Vasiljevic N. Scibior-Bentkowska D. Castanon A. Fiander A. (2013). HPV16 L1 and L2 DNA methylation predicts high grade cervical intraepithelial neoplasia in women with mildly abnormal cervical cytology. Int. J. Cancer. 133, 637–644 10.1002/ijc.28050 23335178 PMC3708123

[B31] Mirabello L. Schiffman M. Ghosh A. Rodriguez A. C. Vasiljevic N. Wentzensen N. (2013). Elevated methylation of HPV16 DNA is associated with the development of high grade cervical intraepithelial neoplasia. Int. J. Cancer 132, 1412–1422 10.1002/ijc.27750 22847263 PMC3493709

[B32] Mirabello L. Sun C. Ghosh A. Rodriguez A. C. Schiffman M. Wentzensen N. (2012). Methylation of human papillomavirus type 16 genome and risk of cervical precancer in a Costa Rican population. J. Natl. Cancer Inst. 104, 556–565 10.1093/jnci/djs135 22448030 PMC3317880

[B33] Nejman D. Straussman R. Steinfeld I. Ruvolo M. Roberts D. Yakhini Z. (2014). Molecular rules governing *de novo* methylation in cancer. Cancer Res. 74, 1475–1483 10.1158/0008-5472.CAN-13-3042 24453003

[B34] Oates N. A. van Vliet J. Duffy D. L. Kroes H. Y. Martin N. G. Boomsma D. I. (2006). Increased DNA methylation at the AXIN1 gene in a monozygotic twin from a pair discordant for a caudal duplication anomaly. Am. J. Hum. Genet. 79, 155–162 10.1086/505031 16773576 PMC1474116

[B35] Piyathilake C. J. Macaluso M. Alvarez R. D. Chen M. Badiga S. Edberg J. C. (2011). A higher degree of methylation of the HPV 16 E6 gene is associated with a lower likelihood of being diagnosed with cervical intraepithelial neoplasia. Cancer 117, 957–963 10.1002/cncr.25511 20945322 PMC3023831

[B36] Schiffman M. Castle P. E. Jeronimo J. Rodriguez A. C. Wacholder S. (2007). Human papillomavirus and cervical cancer. Lancet 370, 890–907 10.1016/S0140-6736(07)61416-0 17826171

[B37] Schiffman M. Wentzensen N. (2013). Human papillomavirus infection and the multistage carcinogenesis of cervical cancer. Cancer Epidemiol. Biomarkers Prev. 22, 553–560 10.1158/1055-9965.EPI-12-1406 23549399 PMC3711590

[B38] Schmieder R. Edwards R. (2011). Quality control and preprocessing of metagenomic datasets. Bioinformatics 27, 863–864 10.1093/bioinformatics/btr026 21278185 PMC3051327

[B39] Sun C. Reimers L. L. Burk R. D. (2011). Methylation of HPV16 genome CpG sites is associated with cervix precancer and cancer. Gynecol. Oncol. 121, 59–63 10.1016/j.ygyno.2011.01.013 21306759 PMC3062667

[B40] Taylor K. H. Kramer R. S. Davis J. W. Guo J. Duff D. J. Xu D. (2007). Ultradeep bisulfite sequencing analysis of DNA methylation patterns in multiple gene promoters by 454 sequencing. Cancer Res. 67, 8511–8518 10.1158/0008-5472.CAN-07-1016 17875690

[B41] Tost J. Gut I. G. (2007a). DNA methylation analysis by pyrosequencing. Nat. Protoc. 2, 2265–2275 10.1038/nprot.2007.314 17853883

[B42] Tost J. Gut I. G. (2007b). Analysis of gene-specific DNA methylation patterns by pyrosequencing technology. Methods Mol. Biol. 373, 89–102 10.1038/nprot.2007.314 17185760

[B43] Turan T. Kalantari M. Calleja-Macias I. E. Cubie H. A. Cuschieri K. Villa L. L. (2006). Methylation of the human papillomavirus-18 L1 gene: a biomarker of neoplastic progression? Virology 349, 175–183 10.1016/j.virol.2005.12.033 16472835

[B44] Turan T. Kalantari M. Cuschieri K. Cubie H. A. Skomedal H. Bernard H. U. (2007). High-throughput detection of human papillomavirus-18 L1 gene methylation, a candidate biomarker for the progression of cervical neoplasia. Virology 361, 185–193 10.1016/j.virol.2006.11.010 17175003 PMC1975683

[B45] Wentzensen N. Sun C. Ghosh A. Kinney W. Mirabello L. Wacholder S. (2012). Methylation of HPV18, HPV31, and HPV45 genomes and cervical intraepithelial neoplasia grade 3. J. Natl. Cancer Inst. 104, 1738–1749 10.1093/jnci/djs425 23093560 PMC3571257

