# Supplementary material for: Characterization of HPV DNA methylation of contiguous CpG sites by bisulfite treatment and massively parallel sequencing—the FRAGMENT approach
Source: Front Genet. 2014 Jun 3;5:150. doi: 10.3389/fgene.2014.00150 (PMC4042685; doi:10.3389/fgene.2014.00150)

Fig S1A. Read counts for each assay

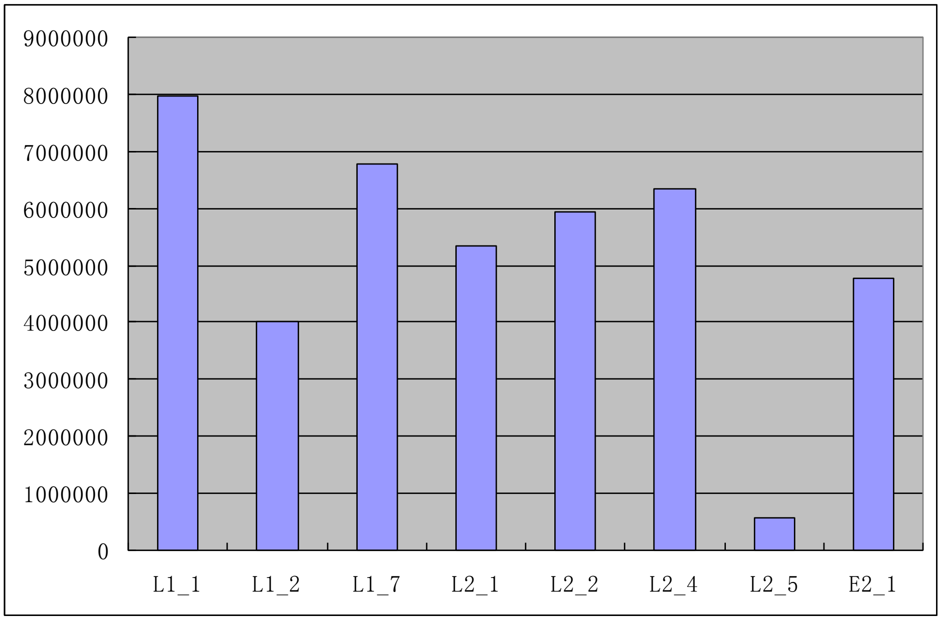

Fig S1B. Read counts for each sample

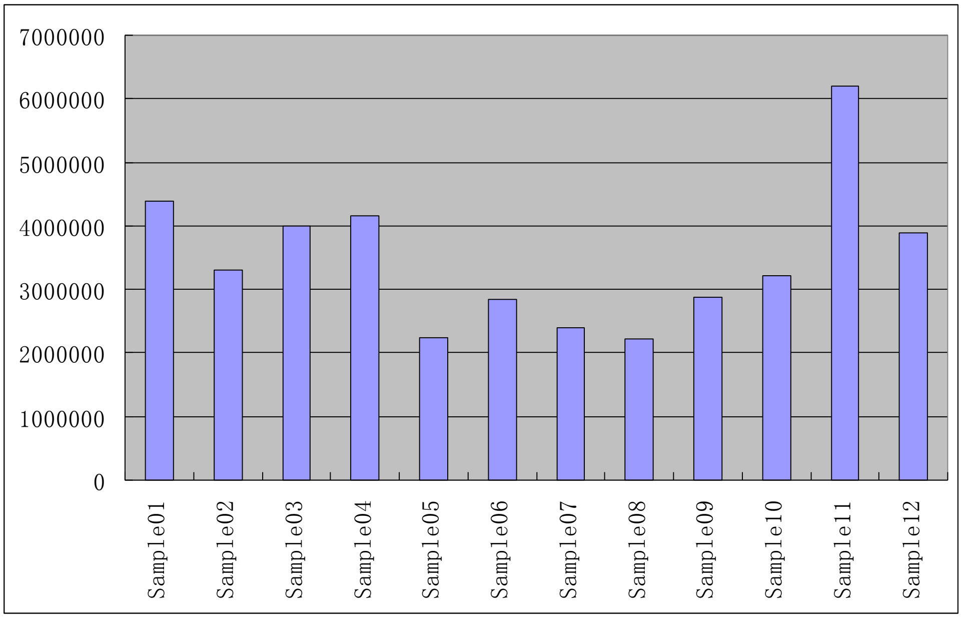

Fig S1C. Read counts for each CpG site

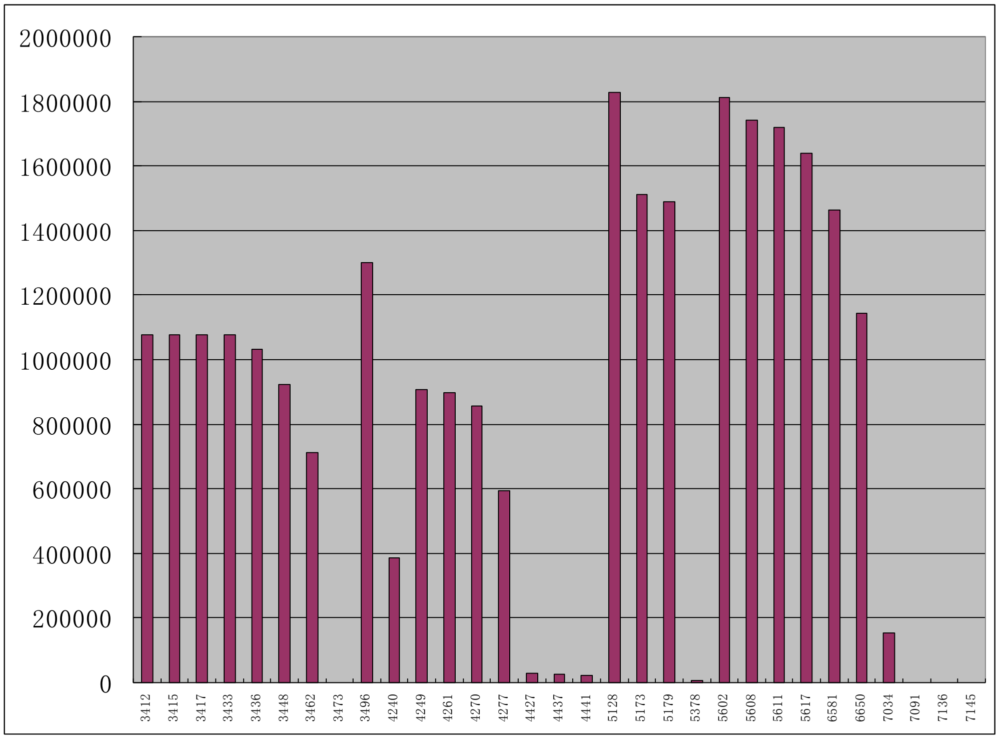

Supplement: Supplementary file 1 [file Presentation1.PDF]
